# Arthroscopic Fixation of a Posterior Cruciate Ligament Femoral Avulsion in an Adult: A Case Report

**DOI:** 10.7759/cureus.83885

**Published:** 2025-05-11

**Authors:** Clevio Desouza, Isteyaque Siddique

**Affiliations:** 1 Orthopaedics, Saifee Hospital, Mumbai, IND

**Keywords:** arthroscopic fixation, femoral avulsion fracture, ligament avulsion, pcl repair, posterior cruciate ligament

## Abstract

Femoral avulsion of the posterior cruciate ligament (PCL) is an exceedingly rare injury pattern in adults, in contrast to the more commonly encountered mid-substance tears or tibial-sided avulsions. We present a rare case of a middle-aged male who sustained a complex multiligamentous knee injury following trauma. Initial imaging revealed avulsion fractures involving both the tibial plateau and medial femoral condyle. Further evaluation with MRI confirmed avulsions of the PCL from its femoral origin and anterior cruciate ligament (ACL) from its tibial attachment, along with posterior root tear of the medial meniscus and associated medial collateral ligament (MCL) injury. The patient was treated with arthroscopically assisted fixation of the PCL via femoral tunnel suturing and suspension device, along with open reduction and internal fixation of the medial epicondyle to restore MCL integrity. Postoperative management included brace immobilization and rehabilitation. At early follow-up, radiographs showed good fragment healing and the patient exhibited restoration of joint stability with clinical improvement. This case emphasizes the importance of early diagnosis through advanced imaging and supports the role of minimally invasive surgical approaches in managing rare, complex ligamentous injuries of the knee.

## Introduction

The posterior cruciate ligament (PCL), known for its strength and stability, is infrequently injured compared to the anterior cruciate ligament (ACL). Most adult PCL injuries involve mid-substance disruptions or tibial avulsions. Avulsion fractures at the femoral origin are rare, particularly in adults, where such injuries typically manifest as soft tissue "peel-off" lesions without bony involvement. Only a handful of adult cases involving femoral bony avulsion have been documented and treated arthroscopically [1-3].

While recent literature reports arthroscopic techniques for various PCL pathologies [4-8], open reduction remains the more conventional approach. This case report describes an exceptionally rare instance of a femoral-side PCL avulsion fracture in a 50-year-old man, managed arthroscopically with successful functional outcomes.

## Case presentation

A 50-year-old male presented with right knee swelling and pain following a motorcycle accident five days prior. There was no distal neurovascular deficit. Physical examination revealed restricted and painful range of motion, joint effusion, and positive anterior and posterior drawer tests. Valgus stress testing suggested medial instability.

**Figure 1:**
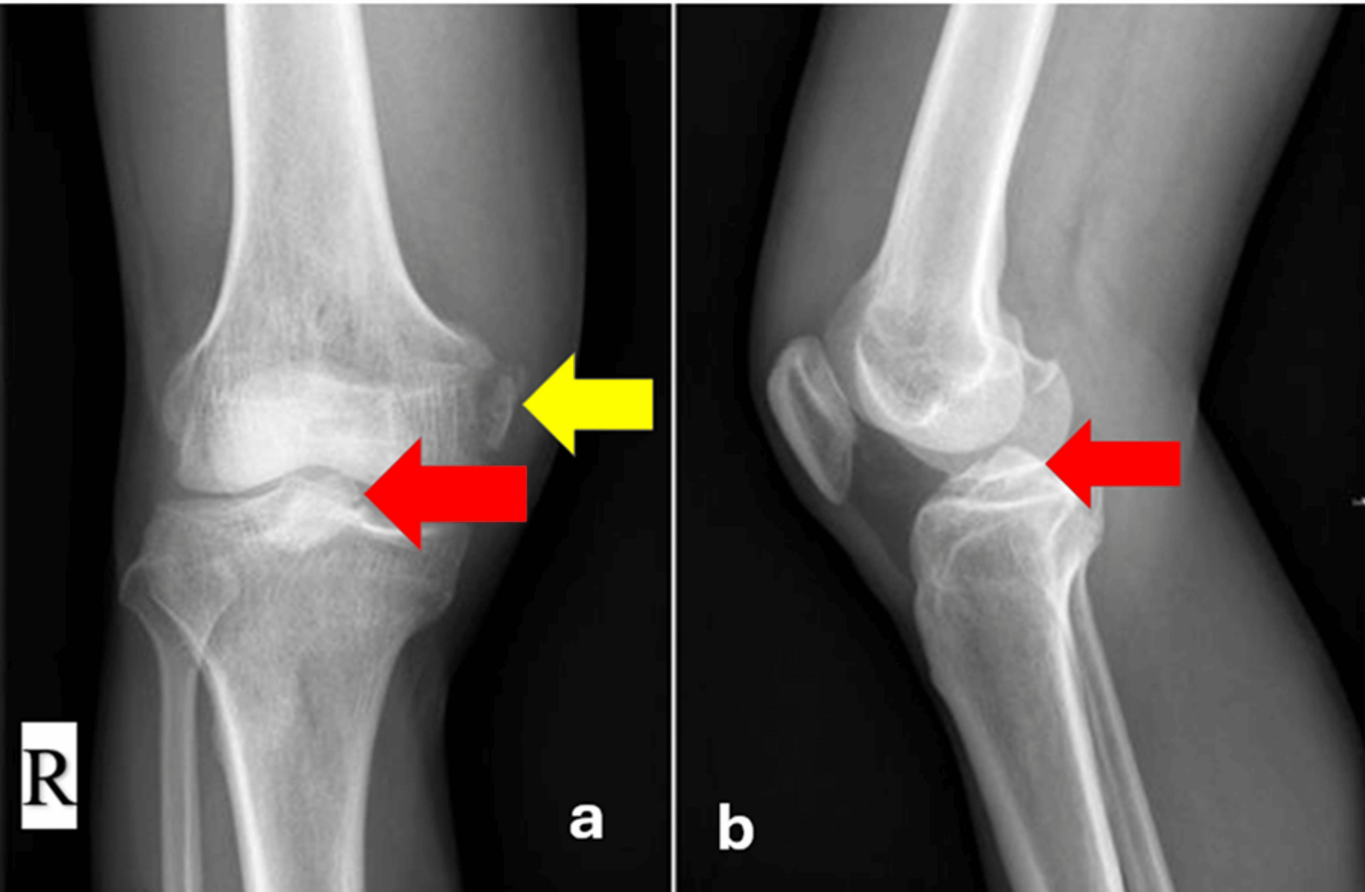
X-ray of the right knee in anteroposterior (a) and lateral (b) views. (a) Anteroposterior view shows avulsion of the anterior cruciate ligament (ACL) from the tibial plateau (red arrow) and avulsion of the medial collateral ligament (MCL) from the femoral condyle (yellow arrow). (b) Lateral view highlights the tibial plateau avulsion of the ACL (red arrow).

Radiographs demonstrated avulsion fractures involving the tibial plateau and the inferior femoral border (Figure 1).

**Figure 2:**
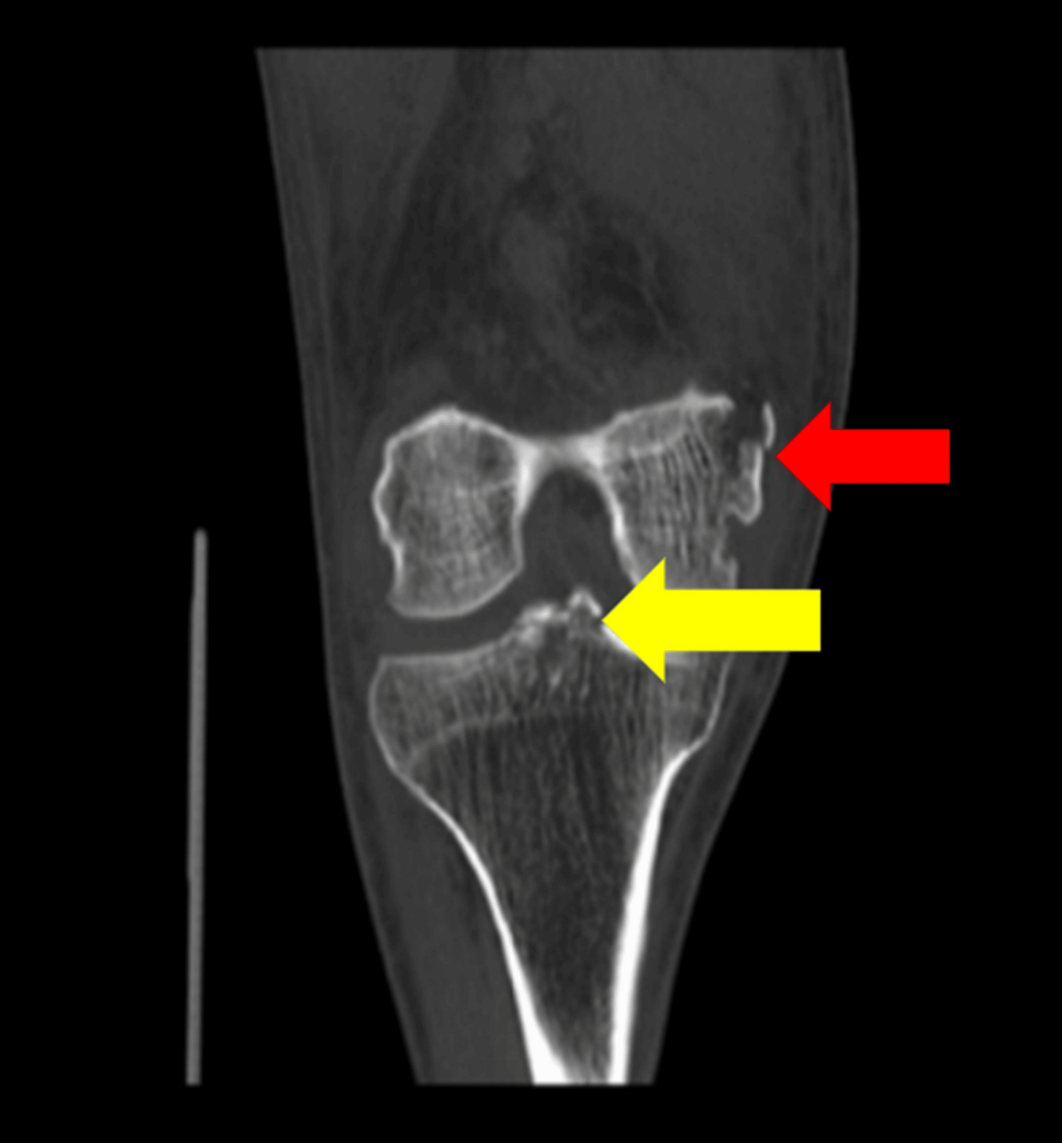
CT scan images of the right knee. The red arrow indicates a fracture of the medial femoral epicondyle, and the yellow arrow highlights an avulsion fracture of the tibial eminence consistent with anterior cruciate ligament (ACL) avulsion.

Computed tomography (CT) scan confirmed bony injury of the femoral medial epicondyle and the tibial plateau (Figure 2).

**Figure 3:**
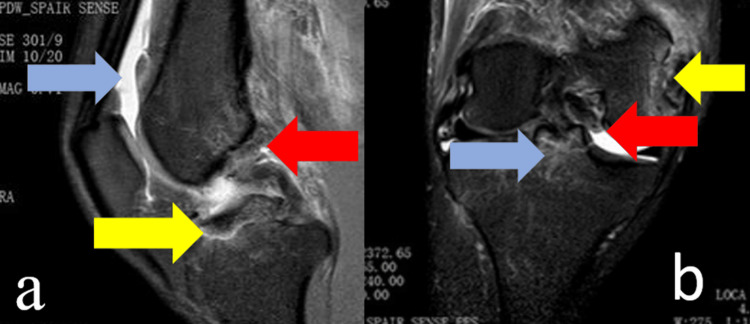
Magnetic resonance imaging (MRI) of the right knee. (a) Sagittal view demonstrates avulsion of the anterior cruciate ligament (ACL) from the tibial insertion (yellow arrow), posterior cruciate ligament (PCL) avulsion from the femoral attachment (red arrow), and joint capsular effusion (blue arrow). (b) Coronal view reveals ACL avulsion from the tibial insertion (blue arrow), posterior root tear of the medial meniscus (red arrow), and avulsion of the medial epicondyle (yellow arrow).

MRI revealed a PCL femoral origin avulsion, an ACL avulsion from the tibial attachment, posterior root tear of the medial meniscus, capsular effusion, and medial epicondyle fracture (Figure 3).

The patient was initially immobilized in an above-knee slab. After swelling subsided, he underwent arthroscopic-assisted fixation of the femoral PCL avulsion.

Surgical technique

**Figure 4:**
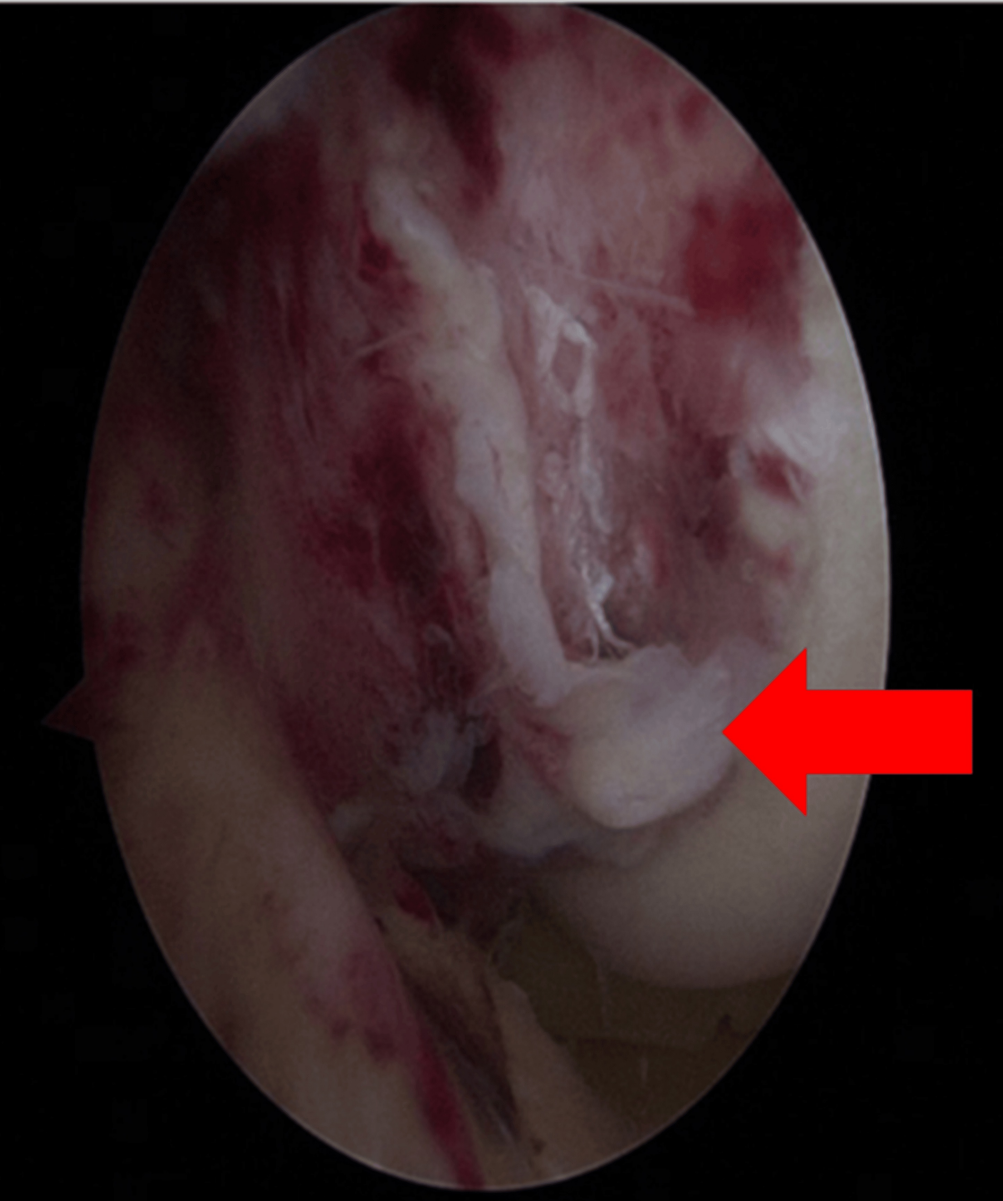
Arthroscopic view of the right knee showing avulsion fracture of the posterior cruciate ligament (PCL) from its femoral attachment (red arrow).

Standard anterolateral and anteromedial portals were used for diagnostic arthroscopy. The ACL appeared intact with a tibial avulsion fragment. The PCL was found avulsed at its femoral attachment, accompanied by partial periosteal stripping and cortical bone involvement (Figure 4).

Debridement was followed by placement of a 4.5-mm absorbable rivet through the anterolateral portal, targeting the medial epicondyle. A suture hook was used to re-anchor the PCL to its femoral footprint. As intraoperative assessment showed persistent laxity, a 4.0-mm femoral tunnel was created, and the PCL was tensioned and fixed using Ethibond sutures, secured with an Endobutton.

Subsequently, open reduction and internal fixation of the medial femoral epicondyle and the MCL tibial attachment were performed using two cannulated screws. Postoperatively, the knee was stabilized in a long leg brace.

**Figure 5:**
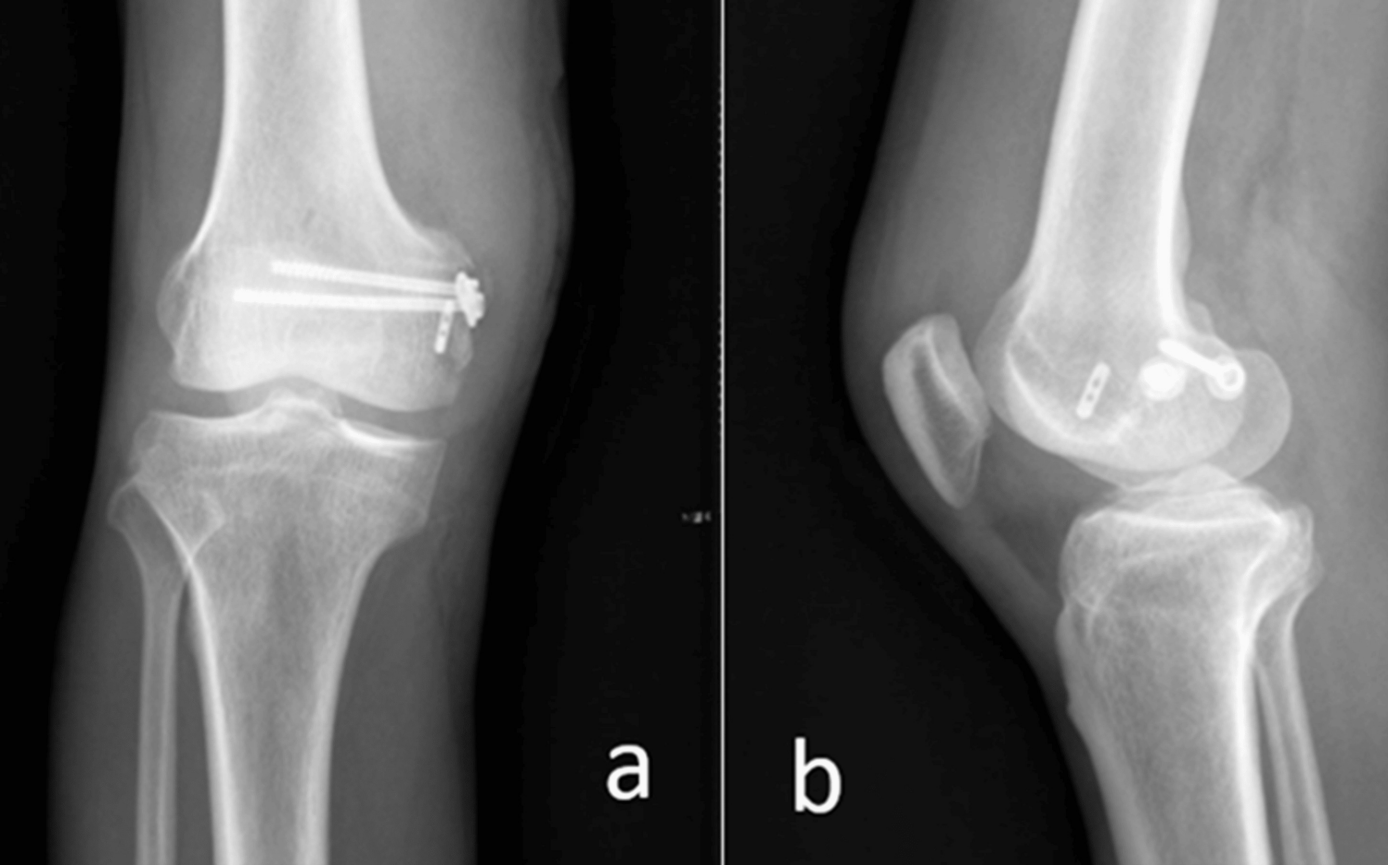
Two-month follow-up X-ray of the right knee. (a) Anteroposterior view and (b) lateral view show satisfactory reduction and healing of the avulsed fragment.

At the two-month follow-up, radiographs confirmed satisfactory reduction of the avulsed fragment (Figure 5). The patient demonstrated improved knee function with active range of motion from 0° to 120°. Clinical stability tests, including anterior and posterior drawer tests, were negative. The absence of instability on clinical examination, restored mobility, and pain-free ambulation indicate favorable early outcomes.

## Discussion

The PCL comprises an anterolateral and a posteromedial bundle, contributing significantly to posterior and rotational knee stability [9,10]. PCL injuries typically involve mid-substance ruptures, and femoral-sided bony avulsions are exceptionally uncommon, especially in skeletally mature individuals [11].

PCL femoral avulsion fractures are more frequently reported in pediatric populations due to relatively weaker physis compared to ligamentous strength [12,13]. These injuries, if unrecognized, may lead to chronic instability and poor functional outcomes [14].

Previous literature includes a few adult cases treated arthroscopically. Park et al. [15] described a transfemoral tunnel technique in a 42-year-old, while Xu et al. [16] reported fixation of a partial osteochondral avulsion using polydioxanone sutures.

Epidemiological data suggest femoral-sided PCL avulsions are exceedingly rare. Gregory et al. [17] noted only one case of femoral-sided PCL injury among 85 patients with associated ligamentous injuries. Earlier reports, such as by O’Donoghue [18] and Drucker and Wynne [19], hint at such injury patterns, although specifics remain unclear. Lee et al. [20] documented a PCL-related osteochondral injury linked to the medial femoral condyle.

The current case describes a high-energy valgus mechanism resulting in a constellation of injuries: femoral MCL avulsion, ACL tibial avulsion, and a femoral PCL avulsion. Arthroscopic-assisted fixation allowed direct visualization and anatomical reduction with minimal invasiveness and early rehabilitation potential.

## Conclusions

Avulsion of the PCL from its femoral attachment is an exceedingly rare injury pattern in adults, often underdiagnosed due to its subtle presentation. Early identification through detailed imaging, including MRI, is crucial for accurate diagnosis and planning surgical intervention. Arthroscopic repair offers a minimally invasive alternative to open surgery, allowing precise anatomical restoration with minimal soft tissue disruption. In this case, we successfully managed a multiligamentous knee injury with femoral PCL avulsion using arthroscopic fixation, demonstrating favorable clinical and radiological outcomes at follow-up. This report adds to the limited literature and reinforces the feasibility of arthroscopic techniques for such rare injuries. Further studies and case series are warranted to establish standardized protocols for managing femoral-side PCL avulsions in adults.
